# In the footsteps of sea stars: deciphering the catalogue of proteins involved in underwater temporary adhesion

**DOI:** 10.1098/rsob.220103

**Published:** 2022-08-17

**Authors:** Morgane Algrain, Elise Hennebert, Philip Bertemes, Ruddy Wattiez, Patrick Flammang, Birgit Lengerer

**Affiliations:** ^1^ Laboratory of Biology of Marine Organisms and Biomimetics, Research Institute for Biosciences, University of Mons, Place du Parc 23, Mons 7000, Belgium; ^2^ Laboratory of Cell Biology, Research Institute for Biosciences, University of Mons, Place du Parc 23, Mons 7000, Belgium; ^3^ Laboratory of Proteomics and Microbiology, Research Institute for Biosciences, University of Mons, Place du Parc 23, Mons 7000, Belgium; ^4^ Institute of Zoology and Center of Molecular Biosciences, University of Innsbruck, 6020 Innsbruck, Technikerstr. 25, Innsbruck 6020, Austria

**Keywords:** Echinodermata, Asteroidea, tube feet, sea star footprint protein, duo-gland adhesive system, proteomics and transcriptomics

## Abstract

Sea stars adhere strongly but temporarily to underwater substrata via the secretion of a blend of proteins, forming an adhesive footprint that they leave on the surface after detachment. Their tube feet enclose a duo-gland adhesive system comprising two types of adhesive cells, contributing different layers of the footprint and de-adhesive cells. In this study, we characterized the catalogue of sea star footprint proteins (Sfps) in the species *Asterias rubens* to gain insights in their potential function. We identified 16 Sfps and mapped their expression to type 1 and/or type 2 adhesive cells or to de-adhesive cells by double fluorescent *in situ* hybridization. Based on their cellular expression pattern and their conserved functional domains, we propose that the identified Sfps serve different functions during attachment, with two Sfps coupling to the surface, six providing cohesive strength and the rest forming a binding matrix. Immunolabelling of footprints with antibodies directed against one protein of each category confirmed these roles. A de-adhesive gland cell-specific astacin-like proteinase presumably weakens the bond between the adhesive material and the tube foot surface during detachment. Overall, we provide a model for temporary adhesion in sea stars, including a comprehensive list of the proteins involved.

## Graphical abstract

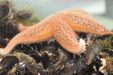

## 1. Introduction

**Figure 1.**
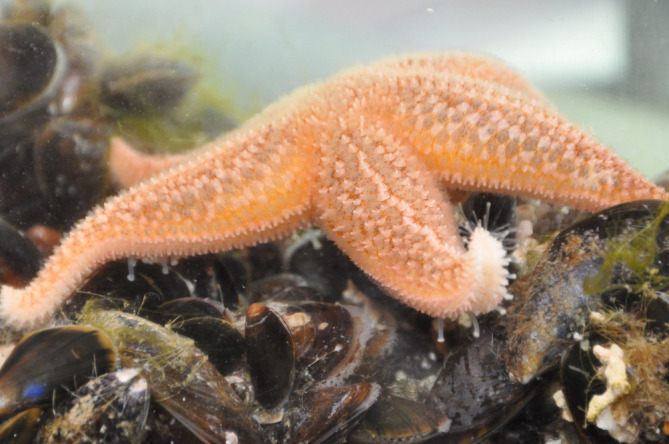
Sea stars use their multiple tube feet to temporarily but strongly attach to many substrata. Here, a sea star of the species *Asterias rubens* crawling over mussels.

Sea stars of the species *Asterias rubens* (Echinodermata, Asteroidea) use specialized adhesive organs, the tube feet, to attach temporarily but strongly to the substratum, as well as to pry open the mussels on which they feed (figure 1). The distal part of the tube foot, called the disc, encloses a duo-gland adhesive system that secretes a blend of proteins forming a bi-layered glue [1–4]. After detachment, the adhesive material is left on the surface as a footprint which consists of a thick structural meshwork layer deposited on a thin homogeneous primer layer [4]. The duo-gland adhesive system comprises two types of adhesive gland cells (adhesive gland cells 1 and 2, AC1 and AC2) and a single type of de-adhesive gland cell (DAC) which can be distinguished by the ultrastructure of their secretory granules [5]. Ultrastructural observations suggest that AC2 secrete the thin homogeneous film that is in contact with the substratum and that AC1 secrete the material forming the thick meshwork on top of the primer layer, probably providing the cohesive interactions between this layer and the tube foot surface [4,6].

The proteins involved in tube foot temporary adhesion in *A. rubens* have been identified by a combined analysis of a tube foot transcriptome and a footprint proteome. Using this approach, the sequences of 34 footprint proteins have been identified [7,8]. Among them, the sea star footprint protein 1 (Sfp1) is the first and only to have been characterized in detail [6]. This large protein of 3853 amino acids (aa) is the second most abundant constituent of the secreted adhesive in *A. rubens*. Sfp1 is auto-catalytically cleaved into four subunits before secretion. In the Sfp1 subunits (named Sfp1 Alpha, Sfp1 Beta, Sfp1 Gamma and Sfp1 Delta), various functional protein domains were identified. These domains are known to mediate protein–protein, protein–carbohydrate and protein–metal interactions [6].

The presence of Sfp1 subunits within the AC1 secretory granules and within the meshwork of the footprints was confirmed by immunofluorescence cytochemistry using anti-peptide antibodies directed against Sfp1 Alpha and Sfp1 Beta. The high abundance and localization of Sfp1 in footprints, and its multi-modular structure based on functional domains indicate that Sfp1 might function as a cohesive protein [6]. Recently, two fragments of Sfp1 were recombinantly produced, comprising most of its functional domains: the C-terminal part of the Beta subunit (rSfp1 Beta C-term) and the Delta subunit (rSfp1 Delta) [9]. The recombinant proteins self-assemble and adsorb on different types of surfaces in the presence of salt ion concentrations found in seawater, forming homogeneous or irregular meshwork coatings [9,10]. The analysis of the adsorption capacities of truncated recombinant rSfp1 Beta C-term proteins on glass highlighted the importance of two functional domains of rSfp1 Beta C-term for its adsorption: the EGF-like domain and an unannotated domain [10]. Furthermore, coatings consisting of rSfp1 Beta C-term or rSfp1 Delta have no cytotoxic effects on HeLa cells and even increase their proliferation [9]. Therefore, recombinant Sfp1 protein coatings could be valuable new materials with potential biomedical applications [9,10].

All footprint protein-encoding transcripts were localized in the tube feet by *in situ* hybridization (ISH) [11]. Out of these 34 sequences, only 22 were exclusively expressed in the secretory cells of the disc adhesive epidermis, while 12 showed additional expression in the stem epidermis. The proteins encoded by the 22 disc-specific transcripts are likely responsible for adhesion in *A. rubens*, but some could alternatively be involved in voluntary tube foot detachment. Concerning the 12 proteins with an additional expression in the stem epidermis, more general functions have been proposed, such as the formation of the glycocalyx-like cuticle. However, full-length coding sequences are not available for the majority of these proteins, making it difficult to identify their function in tube foot temporary adhesion [11].

Prior to this study, only one Sfp1 had been characterized and for many other Sfps only partial sequences were available. Moreover, their cellular site of expression and function are unknown. Here, we characterized a large set of Sfps to provide a more comprehensive picture of the adhesive material formed by *A. rubens*. The previously published sequences of 22 disc-specific proteins were 5′ and 3′ elongated using newly available transcriptomic and genomic data, and the elongated genes were confirmed using polymerase chain reaction (PCR) and Sanger sequencing. We found that some of the partial sequences corresponded to the same gene, reducing the Sfp number. Overall, we obtained 10 full-length and 9 partial Sfps. All Sfps were ascribed to AC1, AC2 or DAC by double fluorescent ISH. The localization of selected proteins in tube foot tissues and footprints was further analysed with polyclonal antibodies. All Sfps were analysed *in silico* in terms of amino acid composition, domain structure and potential post-translational modifications. Taken together, these findings provide insights into the diversity of Sfps and their potential functions.

## 2. Results

### 2.1. Sfp sequence elongation

Sfps are presumably predominantly expressed within the ACs or DACs [3,4,7]. Therefore, we analysed the 22 tube foot transcripts with a corresponding expression pattern [11]. Furthermore, we identified one additional candidate (protein encoded by comp179_c0_seq3) using the data of the footprint mass spectrometry analysis, which was not included in the initial list as it did not fulfil the stringent selection criteria of the study [8]. After confirming its exclusive expression in the adhesive epidermis by ISH (electronic supplementary material, figure S1), it was added as a potential Sfp and was included in all subsequent analyses. This resulted in a list of 23 potential Sfp-coding transcripts. For the majority of the candidate proteins (15/23), the transcriptome sequences did not cover the complete open reading frame (ORF) because the sequences were lacking a start and/or stop codon. To obtain the full-length ORFs, we compared the partial *A. rubens* sequences with similar sequences available in other transcriptomic and genomic resources.

First, we searched for homologues of the partial Sfp ORFs in the transcriptome of the closely related sea star species *Pisaster ochraceus* (http://echinodb.uncc.edu), using the basic local alignment search tool (BLAST) (https://blast.ncbi.nlm.nih.gov/Blast.cgi). As a typical example (see electronic supplementary material, section S2), the sequence of the protein Arub-12 (encoded by transcript comp15624_c0_seq2 [11]) was elongated from 416 up to 747 aa. In that case, the matching sequence found in the *P. ochraceus* transcriptome was longer than our query sequence (electronic supplementary material, figures S2 and S3). We then used the newly identified longer protein sequence for a reciprocal BLAST search in the *A. rubens* tube foot transcriptome [6] (electronic supplementary material, figure S4) and aligned all matching sequences to generate the longer consensus sequence encoding for Sfp10 (see explanation below for the numbering of Sfps). To confirm the accuracy of the elongated sequence, PCRs were performed on *A. rubens* tube foot cDNA and the PCR products were sequenced with Sanger sequencing (electronic supplementary material, table S1). Finally, all elongated sequences were translated to proteins and reanalysed with the original mass spectrometry data [7,8] (electronic supplementary material, figure S5).

In parallel, the partial Sfp ORFs were also elongated using the genome of *A. rubens* (GCF_902459465.1 eAstRub 1.3). We mapped the transcripts from the tube foot transcriptome and the corresponding Illumina sequencing raw reads [8] as well as the coding regions (CDS) predicted by the Sanger Institute to the genome and visualized it using a locally run genome browser. In several cases, this approach revealed that different transcripts with no overlaps were part of one longer transcript. As a typical example (see electronic supplementary material, section S3), transcript comp7100_c0_seq1 coding for Arub-15 and transcript comp19_c0_seq1 coding for Arub-19 [11] were found to be two distant parts of the much longer sequence encoding for Sfp8 (electronic supplementary material, figure S6). Sequences assembled by this method were also confirmed by PCR and subsequent sequencing, as well as by reanalysing mass spectrometry data. Five sequences, however, were located in poorly assembled regions of the *A. rubens* genome (Sfp10, Spf12a/b/c, Sfp13).

Most Sfp sequences could be elongated and confirmed using either one or both of these methods. However, highly repetitive sequences and potential splicing variants made the elongation of some transcripts either complicated or impossible. Overall, we obtained 19 Sfp-encoding sequences, of which 10 comprised the full-length ORF (table 1) (see also 2.4). Several of these sequences were found at the same location on the *A. rubens* genome and were likely either part of the same protein and/or variations of one protein. To highlight this, we named similar or likely connected proteins with the addition of a letter to the same number (e.g. Sfp4a and Sfp4b) (table 1). We thus identified and confirmed 15 different Sfps sequences and named them ‘Sfps’ with ascending numbers as suffix, grouping them according to their expression in the different gland cells (see §2.3). The protein expressed in DAC has an enzymatic domain; therefore, it is not likely to be a structural part of the adhesive footprint but might act in the detachment process. To illustrate the presumed different function of this protein, we named it Astacin-like Sfp (table 1). Genes coding for the Sfps are distributed on seven of the 22 chromosomes of *A. rubens*, and seven Sfp-encoding sequences are closely grouped on one chromosome (Chromosome 6) (table 1).

**Table 1.** Comprehensive list of Sfps in *A. rubens* after *in silico* analysis and sequence elongation. Indicated are the new Sfp names, corresponding transcript IDs from the tube foot-specific transcriptome and names used in previous publications [8,11], completeness of ORF, presence of a signal peptide, protein length in amino acid (aa) and chromosome on which the coding sequence can be found. Sequences that were found at the same location on the *A. rubens* genome and were potentially part or variations of one protein were indicated with the addition of a letter to the number. The numbers in brackets after the transcript ID correspond to the probes used for the double fluorescent ISH experiments (see electronic supplementary material, table S2).

protein name	Genbank accession	transcript ID	corresponding name in Lengerer *et al*. [11]	sequence complete	signal peptide	length (aa)	chromosome bearing the gene
Sfp1	KJ472215.1	comp43_c4_seq1	Sfp1	yes	yes	3853	11
Sfp2	OP067637	comp1654_c0_seq1	Arub-10	yes	yes	3716	11
Sfp3	OP067638	comp17_c0_seq1	Arub-6	yes	yes	1184	6
Sfp4a	OP067639	comp199_c0_seq1	Arub-20	no	no	769	6
Sfp4b	OP067640	comp199_c0_seq5	Arub-1	yes	yes	334	6
Sfp5	OP067641	comp6449_c0_seq1	Arub-3	no	yes	534	6
Sfp6	OP067642	comp1476_c0_seq3(1) comp3966_c0_seq1(2)	Arub-9, -11	yes	yes	2602	20
Sfp7	OP067643	comp1698_c0_seq2(1) comp2480_c0_seq2 comp4570_c0_seq1	Arub-2, -14, -26	no	yes	941	6
Sfp8	OP067644	comp7100_c0_seq1(1) comp19_c0_seq1(2)	Arub-15, -19	yes	yes	1367	6
Sfp9	OP067645	comp362_c0_seq1	Arub-13	yes	yes	1562	10
Sfp10	OP067646	comp15624_c0_seq2(1) comp15624_c0_seq1(2)	Arub-12, -18	no	no	747	?
Sfp11	OP067647	comp179_c0_seq3	—	yes	yes	540	9
Sfp12a/b/c	OP067648 OP067649 OP067650	comp9623_c0_seq4(1) comp9623_c0_seq1(2)	Arub-7, -21	no	no	442/437/425	?
Sfp13	OP067651	comp73892_c0_seq1	Arub-4	no	no	1551	?
Sfp14	OP067652	comp33_c8_seq14(1) comp33_c8_seq19(2)	Arub-5, -16	no	no	229	6
Sfp15	OP067653	comp133_c0_seq4	Arub-8	yes	yes	170	3
Astacin-like Sfp	OP067654	comp204_c0_seq1	Arub-17	yes	yes	445	19

### 2.2. Organization of secretory cells in the tube foot disc

**Figure 2.**
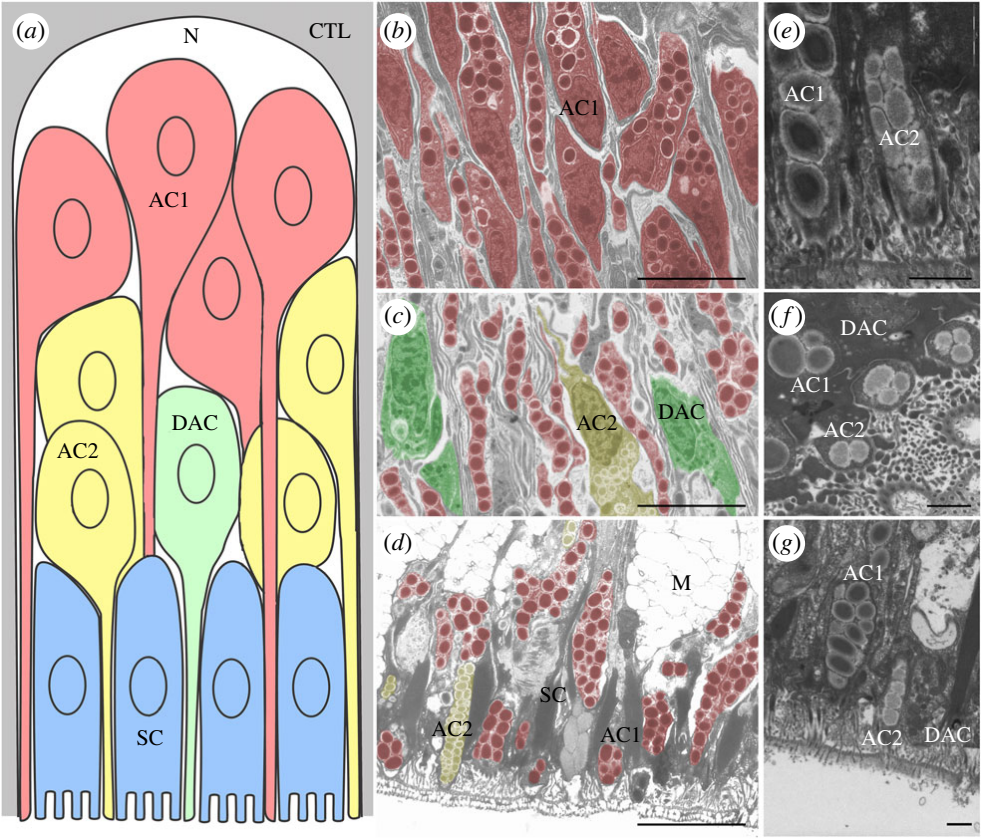
Stratified organization of the gland cells in the tube foot adhesive epidermis. (*a*) Schematic representation of a longitudinal section through a radial epidermal strip located between two adjacent connective tissue laminae (adapted from Hennebert *et al*. [3]; not to scale). The distal surface of the tube foot is at the bottom of the drawing. (*b*–*d*) TEM images taken at the same magnification and showing different areas of the adhesive epidermis corresponding to the areas illustrated in the drawing. Secretory cells are false coloured according to the schematic representation. (*b*) Proximal part showing cell bodies of AC1. (*c*) Middle part showing cell bodies of AC2 and DAC. (*d*) Distal part showing support cells and the apical processes of duo-gland cells (AC1, AC2 and DAC). (*e*–*g*) High magnification on AC1, AC2 and DAC at the distal part of the adhesive epidermis. Scale bars: (*d*) 5 µm, (*e*–*g*) 1 µm. AC1, type 1 adhesive gland cell; AC2, type 2 adhesive gland cell; CTL, connective tissue layer; DAC, de-adhesive gland cell; M, mucus gland; N, nerve plexus; SC, support cell.

As a preamble to the spatial analysis of Sfp expression in tube feet, we performed transmission electron microscopy (TEM) to localize the cell bodies of the ACs and DACs in the disc epidermis (figure 2). Although the adhesive epidermis morphology and ultrastructure have already been reported by Flammang *et al*. [5], the description of the cellular organization was mostly based on the species *Marthasterias glacialis*. In *A. rubens*, the different gland cells can easily be identified thanks to the characteristic ultrastructure of their secretory granules (figure 2*b–g*). The granules of AC1 are large and ellipsoid, enclosing a bundle of parallel electron-dense rods surrounded by a clear cortex. By contrast, the granules of AC2 are medium-size, electron-lucent and their granular core is also surrounded by a clear cortex. Finally, the granules of DAC cells are smaller and contain an electron-dense homogeneous material [5]. AC1 cell bodies were prevalent in the basal part of the epidermis, just adjacent to the basiepithelial nerve plexus (figure 2*a*,*b*). The cell bodies of AC2 were observed closer to the tube foot disc surface, located more distally compared to AC1 cell bodies and intermingled with the long AC1 apical cell processes (figure 2*a*,*c*). The cell bodies of DAC were predominantly located at the area of AC2 cell bodies, but were also identified scattered throughout the whole epidermis. As previously reported [5] DAC were less numerous than AC1 and AC2. All three gland cell types (AC1, AC2 and DAC) possess a single apical process to transport their granules to the distal surface of the adhesive disc (figure 2*d*–*g*). Finally, the cell bodies of support cells (SC) and mucus glands (M) were located in the most distal part of the disc epidermis (figure 2*a*,*d*). The spatial separation of the AC1 cell bodies from the other two gland cells facilitates distinguishing in which gland types the different Sfps are expressed.

### 2.3. Expression of Sfps in adhesive and de-adhesive gland cells

**Figure 3.**
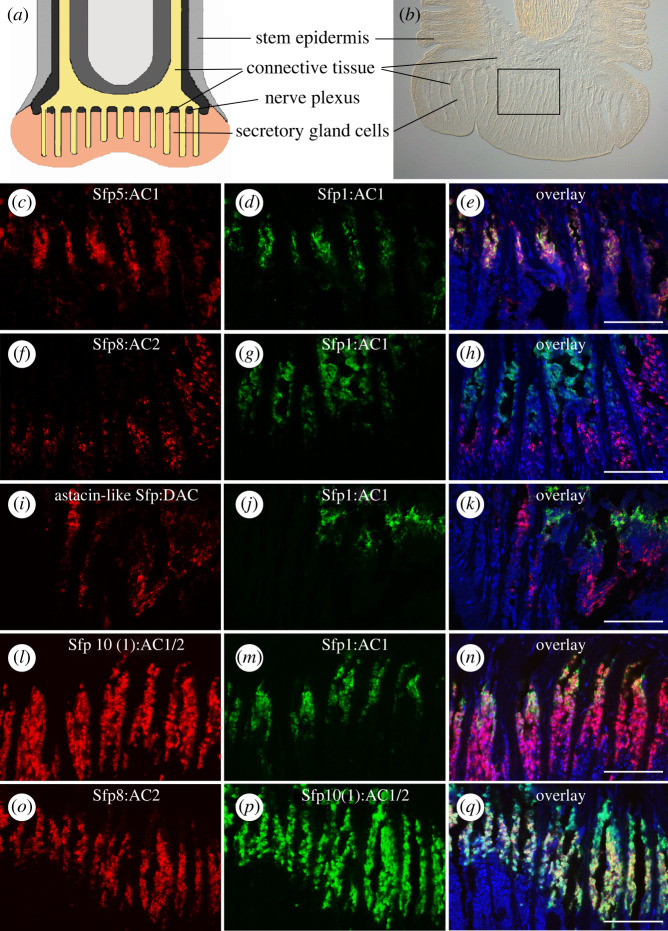
Localization of mRNAs coding for Sfps in the tube foot epidermis of *A. rubens* visualized by double ISH. (*a*) Schematic drawing and (*b*) bright field picture of a longitudinal tube foot section. Boxed area indicates the approximate area of double ISH images. (*c–n*) Localization of mRNAs coding for the AC1-specific protein Sfp1 (*d*,*g*,*j*,*m*; in green) and those coding for AC1-specific Sfp5 (*c*,*e*), AC2-specific Sfp8 (*f*,*h*), DAC-specific Astacin-like Sfp (*i*,*k*) and AC1/2 expressed Sfp10 (*l*,*n*) (in red). (*o*–*q*) Localization of the transcripts coding for the AC2-specific Sfp8 (in red) and AC1/2 expressed Sfp10 (in green). The nuclei were stained with DAPI (in blue). When different probes were available for a single Sfp, they were identified by a number between brackets (e.g. Sfp8(1); see electronic supplementary material, table S2). Schematic drawing in (*a*) modified after Santos *et al*. [12]. Scale bars: 50 µm.

Based on detailed ultrastructural investigations, the two adhesive gland cells were proposed to contribute differently to the two layers of the footprint [4], with AC2 secreting the homogeneous layer in contact to the surface and AC1 forming the meshwork on top [4,6]. Therefore, it is functionally relevant to investigate which proteins are produced by which cell type and we would expect Sfps expressed in AC2 to have an adhesive function and those expressed in AC1 a cohesive function. Several Sfps have been identified in *A. rubens* footprints (see §2.1), but only Sfp1 has been unambiguously associated with the secretory cell type AC1. The localization of Sfp1 in AC1 granules was shown in immuno-TEM with specific antibodies [6]. However, as the production of specific antibodies for all Sfps is not cost-effective and carries no guarantee of success, we decided to instead perform double ISH experiments to compare the expression of all Sfp-coding transcripts with the expression of Sfp1 and among each other. We used the ISH probes corresponding to the original 23 transcripts for this purpose [11]. Therefore, for several Sfps more than one ISH probe was available (indicated in the figures by an additional number in brackets after the Sfp name). As many double ISH patterns were similar, only those showing the expression of Sfps representative of each cell type are shown in figure 3. All double staining results can be found in electronic supplementary material, figures S7–S10. To help interpret the staining results, a schematic drawing and a bright field image of an *A. rubens* tube foot section is presented in figure 3*a*,*b*. The transcripts coding for Sfp2, Sfp3, Sfp4a, Sfp4b, Sfp5 and Sfp6 were exclusively co-localized with the transcript coding for Sfp1 and therefore located in AC1 (figure 3*c*–*e* and electronic supplementary material, figure S7). The labelling obtained for the transcripts coding for three other Sfps showed no overlap with the labelling obtained with the transcript coding for Sfp1 and therefore localized to AC2 or DAC (electronic supplementary material, figure S8). AC2 are more numerous than DAC [5] and, based on the relative number of labelled cells, the transcripts coding for Sfp7 and Sfp8 could be assigned to AC2 (figure 3*f*–*h*; electronic supplementary material, figure S8) and the one coding for Astacin-like Sfp to DAC (figure 3*i*–*k*). The localization of the transcript coding for Astacin-like Sfp within DAC showed no overlap with an AC2-specific transcript (coding for Sfp8) (electronic supplementary material, figure S8*g*–*i*). Finally, transcripts coding for Sfp10, Sfp11, Sfp12a, Sfp12b, Sfp12c, Sfp13, Sfp14 and Sfp15, were localized both within AC1 and AC2 (figure 3*l*–*n*; electronic supplementary material, figures S9 and S10). To confirm the localization of these transcripts in both adhesive gland cells, we compared their localization with that of the AC2-specific transcript Sfp8 (figure 3*o*–*q*) in addition to comparing them to the AC1-specific Sfp1.

### 2.4. *In silico* protein characterization of Sfps

**Figure 4.**
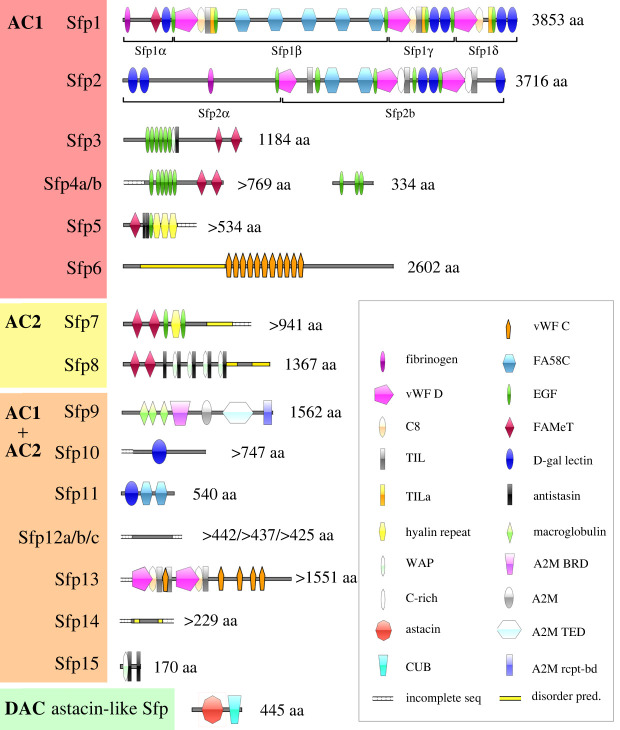
Conserved domain architecture of Sfps. The proteins were grouped according to their secreting cell: AC1 only, AC2 only, both adhesive gland cell types (AC1 + AC2), or DAC. AC1, type 1 adhesive gland cell; AC2, type 2 adhesive gland cell; DAC, de-adhesive gland cell; A2M, alpha-2-macroglobulin; A2M BRD, alpha-2-macroglobulin bait region domain; A2M rcpt-bd, alpha-2-macroglobulin receptor-binding domain; A2M TED, alpha-macroglobulin-like thioester domain; CUB, complement C1r/C1 s, uEGF, BMP1 domain; C8, domain of eight conserved cysteine residues; disorder pred., areas of predicted disorder with no stable secondary structure; D-Gal lectin, D-Galactose binding lectin domain; EGF, epidermal growth factor-like domain; FAMeT, Farnesoic acid O-methyltransferase domain; FA58C, coagulation factor 5/8 type C-terminal domain; incomplete seq, start and/or end of sequence is unknown; TIL/TILa, trypsin inhibitor-like cysteine-rich domain; vWF C, von Willebrand factor type C domain; vWF D, von Willebrand factor type D domain; WAP, whey acidic protein domain.

The large majority of Sfps comprise functional protein domains (figure 4). Sfp1 has been shown to be part of the cohesive meshwork layer proximal to the disc and is solely expressed in AC1 [6]. Similar to Sfp1, we identified another AC1-specific protein, Sfp2, with comparable protein domains and one auto-catalytic cleavage site (GPDH sequence) that should lead to the formation of two subunits (Sfp2α and Sfp2β). Additionally, three proteins with repetitive EGF-like domains were found to be exclusively expressed in AC1 glands (Sfp3, Sfp4a and Sfp4b). Sfp4b is also rich in serine and glycine residues (electronic supplementary material, table S3). Four AC1-specific Sfps (Sfp1, Sfp3, Sfp4a and Sfp5) contain farnesoic acid O-methyl transferase (FAMeT) domains. Finally, in this gland cell type, Sfp5 possesses 3 hyalin repeat domains while Sfp6 comprises a disordered domain rich in glutamate followed by repetitive von Willebrand factor type C domains (vWF C) (figure 4, electronic supplementary material, table S3). All the Sfps produced exclusively by AC1 are characterized by a high cysteine content (electronic supplementary material, table S3).

The two AC2-specific proteins (Sfp7 and Sfp8) both contain two FAMeT domains. Sfp7 additionally comprises two EGF-like and one hyalin domain, whereas Sfp8 also contains several repetitions of whey acidic protein (WAP) and antistasin domains. Due to their expression in AC2, these two Sfps are likely secreted onto the substratum surface and presumably form the homogeneous primer layer [4].

Among the proteins expressed in both adhesive gland cell types, one alpha-macroglobulin-like protein (Sfp9) was found. There were also two proteins (Sfp10 and Sfp11) containing carbohydrate-binding domains (galactose-binding or coagulation factor 5/8 C-terminal domain (FA58C)), and one (Sfp13) comprising vWF D and vWF C domains. Several proteins (Sfp12a/b/c, Sfp14) did not contain any annotated protein domains, which might be artefactual, as only partial sequences are available. The three variants of Sfp12 are variations of a repetitive protein sequence, which is rich in threonine residues and predicted to be highly O-glycosylated relative to their size (electronic supplementary material, table S3).

### 2.5. Localization of selected Sfps on tube foot sections and footprints

**Figure 5.**
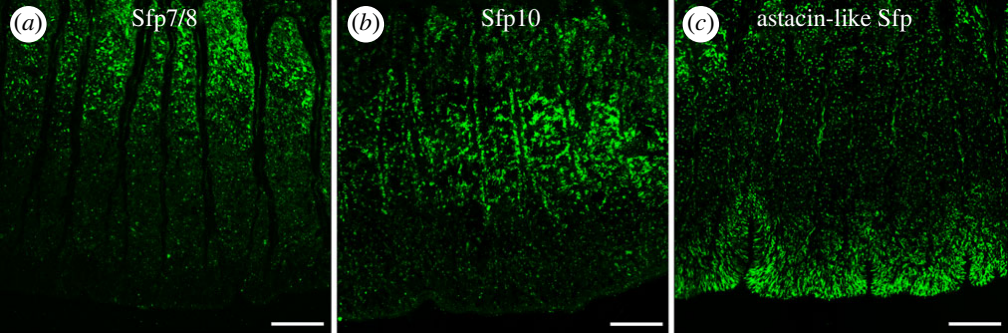
Immunofluorescence localization of selected Sfps in tube foot sections of *Asterias rubens*. Tube foot longitudinal sections were labelled with antibodies directed against (*a*) Sfp7/8, (*b*) Sfp10 and (*c*) Astacin-like Sfp (labelling in green). Scale bars: 50 µm.

To localize selected proteins in tube foot tissues and in footprints (i.e. before and after secretion, respectively), three polyclonal antibodies were produced. The first one was directed against peptides from Sfp7/8 (specific to AC2), the second one against Sfp10 (expressed in both AC1 and AC2) and the third against Astacin-like Sfp (specific to DAC) (target peptide sequences in electronic supplementary material, section S4). The Sfp7/8-specific antibody was directed against a peptide present in both proteins and therefore does not allow us to distinguish between them. All three antibodies reacted with protein bands in western blots of tube foot and footprint proteins (electronic supplementary material, figure S11). On tube foot sections, all three antibodies reacted with granular structures within the gland cells of the duo-gland adhesive system, as expected (figure 5). However, it is difficult to establish a clear correlation between immunolabelling and the labelling obtained in ISH because of the differences in targeted molecules (protein and mRNA, respectively) and in their distribution (cell body around the nucleus versus secretory granules throughout the cell). Moreover, immunolabelling might also be influenced by protein maturation processes occurring along the secretory pathway.

**Figure 6.**
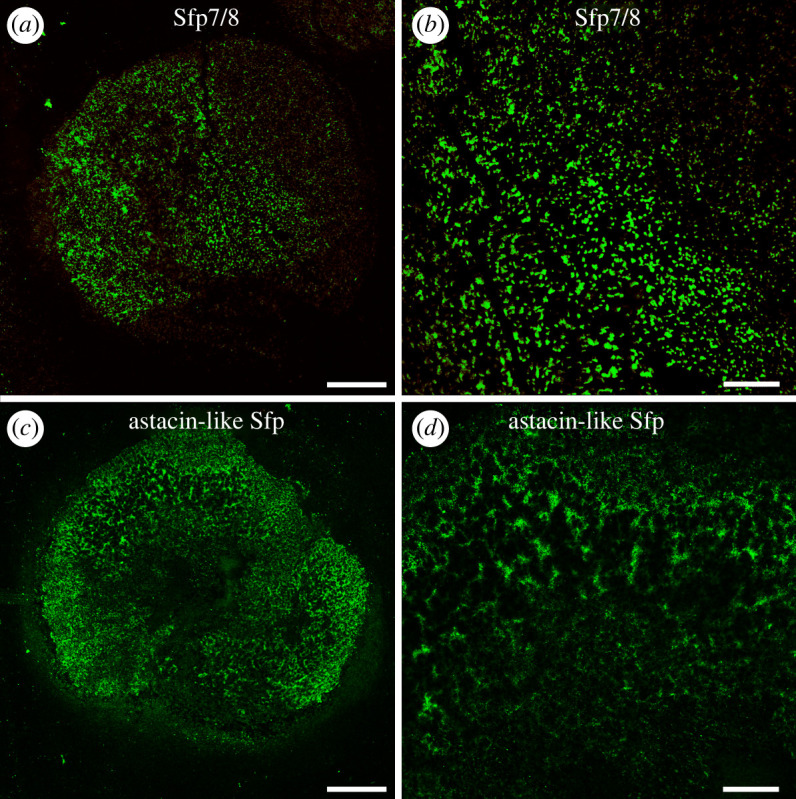
Immunofluorescence localization of selected Sfps in the footprints of *Asterias rubens*. Footprints were labelled with antibodies directed against Sfp7/8 (*a*,*b*) and Astacin-like Sfp (*c*,*d*). Scale bars: 100 µm (*a*,*c*) and 30 µm (*b*,*d*).

**Figure 7.**
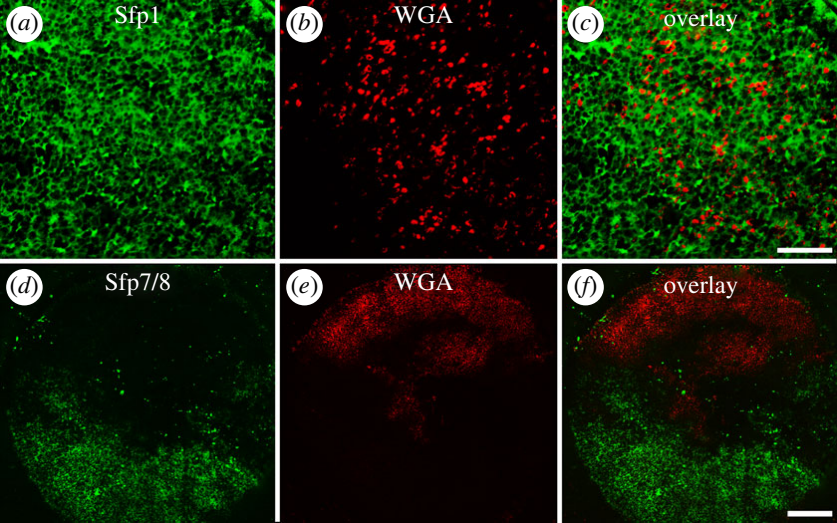
Double immuno- and lectin-labelling on footprints of *Asterias rubens*. Footprint labelled with antibodies directed against Sfp1β (*a*), with the lectin WGA (*b*) and overlay image (*c*). Footprint labelled with antibodies directed against Sfp7/8 (*d*), with WGA (*e*) and overlay image (*f*). Scale bars: 20 µm (*a*–*c*) and 100 µm (*d*–*f*).

In the footprints, the anti-Sfp7/8 antibody-labelled structures appeared as individual dots (figure 6*a*,*b*). Immunolabelling with antibodies directed against Sfp10 appeared blurry and were difficult to interpret (electronic supplementary material, figure S12). With the anti-Astacin-like Sfp antibody, the labelling was present on the whole footprint, forming a network (figure 6*c*,*d*), but appeared different from the meshwork pattern obtained with the anti-Sfp1β antibody (compare figures 6*d* and 7*a*; see also [6]).

In a previous study, commercially available lectins have been used to detect glycans present in the secreted footprints and foot sections [13]. Wheat germ agglutinin (WGA) led to a dot-like labelling [13] resembling the labelling obtained with the anti-Sfp7/8 antibody (figure 6*a*,*b*). To test if these two labellings overlap, we performed double labelling experiments with the lectin WGA and either anti-Sfp1β or anti-Sfp7/8 antibodies. Double labelling with anti-Sfp1β antibodies (in green) and WGA (in red) showed that their target molecules co-occur in the same areas of footprints, the glycans detected with WGA being located in between the meshwork containing Sfp1 (figure 7*a*–*c*). On the contrary, the anti-Sfp7/8 (in green) and WGA (in red) labelling were always completely separated within a single footprint (figure 7*d*–*f*). When an area of the footprint was labelled with the anti-sfp7/8 antibody, the labelling with WGA was missing and *vice versa* (figure 7*f*). According to Hennebert *et al*. [4], the thickness of the adhesive layer varies between different areas in a same footprint, giving different aspects to the adhesive material. To test if the labelling pattern we observed could be due to footprint areas with different thicknesses, we changed our footprint sampling technique. Instead of letting the animals attach strongly to a glass slide for prolonged time, we simply let them walk over glass slides. This way, less material was secreted, and the resulting footprints were thinner. In these thinner footprints, there was an extensive labelling covering the whole footprint with the anti-Sfp7/8 antibody but no labelling with WGA (electronic supplementary material, figure S13).

## 3. Discussion

Temporary adhesion can be defined as a reversible attachment process [14]. Many marine and freshwater organisms have developed temporary adhesion systems that show complexity at different length scales [15]. At the cellular level, adhesive secretions may be produced by one or more secretory cell types while, at the molecular level, the number and structure of adhesive proteins vary greatly from one taxonomic group to another [16,17]. In the present study, we have deciphered the complex cellular and molecular organization of the temporary adhesive system of *A. rubens*.

### 3.1. Cellular organization of the duo-gland adhesive system

The cellular basis of marine temporary adhesion is often a duo-gland adhesive system [1,18]. The simplest duo-gland adhesive systems have been described in free-living flatworms, like the marine *Macrostomum lignano* [19]. In *M. lignano*, the adhesive tail plate consists of multiple adhesive papillae, with each comprising one adhesive gland cell, one releasing (de-adhesive) gland cell and one anchor cell [19]. By contrast to these tiny flatworms, echinoderms are commonly several centimetres in size and rely on millimetre-sized appendages, the tube feet, for adhesion. Although some echinoderm tube feet bear scattered sensory–secretory complexes resembling flatworm adhesive papillae, the tube feet of many sea urchins and sea stars end in a single large adhesive disc [2,20]. The tube foot adhesive disc contains high numbers of ACs and DACs uniformly distributed within a framework of support cells, mucus gland cells, sensory cells and load-bearing connective tissue. In sea urchins, a single gland cell type produces the adhesive secretion [21,22]. In contrast, in many sea stars two adhesive cell types can be distinguished [5,23]. The reason for the evolution of two types of adhesive cells in most sea stars versus one type in sea urchins is currently unknown.

Contrary to the cellular organization in the spiny sea star *M. glacialis* [5] or the starlet cushion sea star *Asterina gibbosa* [23], the cell bodies of AC1 and AC2 in *A. rubens* were spatially separated within the adhesive epidermis. As was the case in the flatworm *M. lignano* in which a similar spatial separation is observed between adhesive and releasing gland cell bodies [19,24]. This spatial segregation facilitated gene expression analysis with ISH and thus the localization of the production of the different Sfps in one or the other of the adhesive gland cells (figure 3).

### 3.2. Molecular complexity of the duo-gland adhesive system

In the sea star *A. rubens*, we identified a set of 16 Sfps (19 including variants). The different Sfps have been named according to their expression in the different gland cells highlighted by double fluorescent ISH. Sfp1-6 are exclusively expressed in AC1, Sfp7 and Sfp8 exclusively in AC2, and Sfp9–15 were found to be expressed in both AC1 and AC2. The last Sfp, Astacin-like Sfp, was found in DAC. New antibodies were generated against Sfp7/8, Sfp10 and Astacin-like Sfp, which all labelled the adhesive epidermis. Unfortunately, it was not possible by this method to confirm the cellular origin of these three Sfps as the labelling was restricted to some areas of the gland cells and did not extend throughout the cells (figure 5). This effect has been previously observed with lectin histochemistry [13]. Most likely, it is an artefact due to differences in the accessibility of the different epitopes according to the maturation of the adhesive material during its transport from the base to the apex of the adhesive gland cells [5]. Moreover, even with the available *A. rubens* genome (eAstRub 1.3), the complete ORFs of five Sfps could still not be determined. For Sfp4a, Sfp7 and Sfp14, multiple splice variants were predicted and the correct ORF remained unsolved. The sequences of Sfp10, Sfp12 and Sfp13 were found with low identity on genomic scaffolds that could not be assigned to a chromosome.

To date, all characterized aquatic adhesives are composed of multiprotein complexes [25]. Each individual protein of these complexes is supposed to have a specialized function within the adhesive layer, including surface coupling (adhesion), structural support (cohesion) and protection against degradation [26]. The best-characterized temporary adhesion system is the one of macrostomid flatworms. It is based on two large adhesive proteins only (ap1 and ap2) [24,27]. In *M. lignano*, the glycosylated Mlig-ap2 is in contact with the surface while Mlig-ap1 is providing internal cohesion [27]. In comparison, 21 proteins are potentially involved in the temporary adhesion of the hydrozoan *H. magnipapillata* [28], and 16 in the sea urchin *Paracentrotus lividus* [29]. However, these numbers must be confirmed as full-length sequences are not available yet for many of these proteins.

### 3.3. Role of the different Sfps within the adhesive

Based on their specific expression within the adhesive gland cells and on their functional domains, we propose that the identified Sfps serve different functions during attachment. However, assigning a specific role to each protein is speculative at the moment and it is likely that several proteins share a function and/or serve more than one function. The complex repartition of Sfps between AC1 and AC2 (AC1-specific, AC2-specific and AC1/2 co-expressed) might be linked to Sfp storage within the secretory granules and influence the protein distribution in the footprints. Indeed, TEM observations of attached tube feet indicated convincingly that AC1 secrete an electron-dense material forming the cohesive fibrous meshwork of the footprint, while AC2 produce a moderately electron-dense material forming the homogeneous primer layer of the footprint [4]. Both secretions are embedded in a loose electron-lucent material [4], which presumably comes from the electron-lucent rim of the secretory granules.

Immunolabelling in light and electron microscopy demonstrated that Sfp1 is located in the fibrous core of the secretory granules stockpiled by AC1 and that after secretion it makes up the structural scaffold of the adhesive footprint [6]. Like Sfp1, Sfp2–6 are exclusively expressed in AC1 and could, therefore, be involved in the formation of the cohesive meshwork. Sfp2 is very similar to Sfp1 and to other potential cohesive proteins identified in different flatworm species (ap1), sea urchins (Sfp1-like) and limpets (P-vulgata_3), which all contain von Willebrand factor (vWF) domains consisting of vWF D, C8 and TIL [17,24]. vWF is known to be involved in protein multimerization to form compact filaments [30].

Sfp3, Sfp4a and Spf4b contain multiple calcium-dependent epidermal growth factor-like (EGF) domains. The presence of multiple tandem repeats of EGF domains is another common feature of marine adhesives which has been found in proteins from mussels [31], limpets [32], sea urchins [29] and sea anemones [33]. The flatworm protein Mlig-ap1 contains 17 EGF domains and is assembled into fibres when secreted [27]. The prevalence of repetitive EGF-like domains in proteins expressed in AC1 could, therefore, be linked to the fibrous meshwork formation. In recombinantly produced Sfp1β, the EGF-like domain was found to play an important role for the protein adsorption to glass [10].

Sfp5 contains hyaline repeat domains. In sea urchins, the large glycoprotein hyalin, consisting of hyaline repeat domains, forms an extraembryonic matrix which serves as a cell adhesion substrate during early development [34,35]. Sfp6 contains vWF type C repeat domains which is similar to kielin/chordin-like protein, an extracellular matrix protein.

Known surface-binding proteins in mussels, like mussel foot proteins (Mfp) 3 and 5 are disordered, which is required to optimize surface interactions [36]. With this in mind, we identified regions of predicted disorder in Sfp7 and Sfp8, the potential surface-binding Sfps exclusively expressed in AC2. Disorder predicted sequences do not possess stable secondary and/or tertiary structures [37]. Many proteins possessing disordered regions (proteins that do not contain sufficient hydrophobic amino acids to mediate cooperative folding) are able to interact with various partners like themselves, other proteins, membranes, nucleic acids and metal cations [38–40]. Additionally, the AC2-specific Sfps possess FAMeT domains. In arthropods, farnesoic acid O-methyltransferase catalyses the formation of methyl farnesoate from farnesoic acid and is involved in growth and moulting [41,42]. In other organisms, FAMeT domains have been reported in proteins involved in the biomineralization process in molluscs [43,44] but their function is not known. FAMeT domains have not been described in any other adhesive protein from aquatic organisms and might be sea star specific. Interestingly, they were also detected in four Sfps that were exclusively expressed in AC1. However, the role of this domain in the sea star adhesive footprint remains unknown.

Sfp8 comprises several WAP and antistasin domains. Both may provide stability in harsh physical conditions through multiple intramolecular disulfide bridges [45]. These domains are found in serine proteinase inhibitors which may play a role in antibacterial activity [46–49]. These domains were also identified in Sfp3 and Sfp15. A protein with high similarity to Sfp15 was also described in the adhesive of the limpet *P. vulgata* (P-vulgata_12) [32]. In the mussel *Perna viridis*, a byssal protein with antistasin domains has been identified that may be able to bind metals via motifs similar to metallothioneins [50,51].

Although domain analysis cannot confirm the role of Sfp7 and Sfp8 in surface binding, this proposed function is corroborated by the immunolabelling of footprints with the anti-Sfp7/8 antibodies. When footprints are thin (e.g. when sea stars simply walk along the substratum) Sfp7/8 can be detected between the meshwork. When footprints are thicker (e.g. when sea stars need to counteract hydrodynamic forces), more cohesive material is secreted and the thick meshwork precludes the detection of Sfp7/8. Therefore, the immunoreactivity pattern observed can be explained by the presence of Sfp7 and Sfp8 in the homogeneous primer layer beneath the meshwork.

Sfp9–15 were found to be expressed in both AC1 and AC2. It is, therefore, tempting to hypothesize that they could originate from the electron-lucent outer rim shared by both types of granules and form the electron-lucent binding matrix of the footprint after secretion. In the flatworm *M. lignano*, the two adhesive proteins are stored in separate areas of the adhesive gland cell secretory granules, with Mlig-ap1 forming the electron-dense inner core and Mlig-ap2 the more lucid outer rim [27]. Like Mlig-ap2, two Sfps found in both adhesive cell types, Sfp12 and Sfp14, are predicted to be highly glycosylated. Glycosylated mucin-like proteins are characterized by an electron-lucent appearance in TEM [52]. Interestingly, other Sfps, such as Sfp 10 and Sfp11 but also Sfp1 and Sfp2 secreted by AC1, possess glycan-binding lectin domains. The presence of both glycans and glycan-binding lectin domains in footprints might indicate that sugar–protein interactions are involved in their non-covalent cross-linking.

Sfp9 is an alpha-2-macroglobulin-like protein. Similar proteins have been discovered in the adhesive secretions of barnacle cyprids [53], limpets [32], sea urchins [29] and ascidians [54]. They are, therefore, a common feature of aquatic bio-adhesives [17]. Alpha-2-macroglobulin-like proteins share the function of binding various proteins and peptides [55] and Sfp9, therefore, also potentially mediates non-covalent cross-linking within the footprints.

### 3.4. The Astacin-like Sfp might facilitate detachment

Animals with a duo-gland adhesive system produce both a sticky and a de-adhesive substance to perform their attachment and detachment cycles. This de-adhesive substance can either compete with the adhesive proteins for binding sites on the animal's surface (competition model) or act enzymatically to digest these links (enzymatic model) [3,14]. In sea stars, the competition model has been proposed by Thomas & Hermans [56] in the species *Leptasterias hexactis*. In *A. rubens*, the enzymatic model has been suggested by Flammang *et al*. [3], and recently a proteinase was identified in the footprint proteome [8]. In this study, we showed that this proteinase is indeed expressed exclusively in DAC. The presence of a conserved peptide motif (HExxHxxGxxH) indicated it contains an astacin-like domain [57]. We, therefore, named this proteinase Astacin-like Sfp. Astacins are extracellular metalloproteinases with manifold functions, ranging from embryo hatching to developmental processes and tissue differentiation [58]. Astacin-like Sfp also contains a CUB domain (for complement C1r/C1 s, Uegf, Bmp1), a domain which can allow protein–protein and enzyme–substrate interactions via carbohydrates [59]. Immunolabelling of Astacin-like Sfp indeed indicates that it remains bonded on the surface of the footprint after tube foot detachment. The exclusive expression of Astacin-like Sfp in DAC, as well as its presence on the surface of the footprints, both indicate that this proteinase might facilitate the detachment process in *A. rubens*.

## 4. Conclusion

Fuelled by previous research and by integrating the results provided in this study, the complex temporary adhesion in the sea star *A. rubens* can be better understood. Here, we identified a catalogue of Sfps expressed in adhesive gland cells. The secretions of AC2 are probably in direct contact with the substratum [4]. We identified two proteins, Sfp7 and Sfp8, which are exclusively expressed in AC2, and antibody staining confirmed their presence in the primer film of the footprints. Six Sfps, including the well-characterized Sfp1 [6], were found to be AC1 specific. Based on their expression and functional domains, they presumably form the cohesive meshwork. Sfp9–15 were found to be expressed in both AC1 and AC2 and probably form a binding matrix within the footprints. For detachment, Astacin-like Sfp is secreted by DAC, weakening the bond between the adhesive layer and the tube foot disc surface. The tube foot can then detach more easily, leaving the adhesive material on the substratum as a footprint.

## 5. Material and methods

### 5.1. Collection and maintenance of sea stars

Individuals of *Asterias rubens* [60] were collected intertidally in Audresselles (Pas-de-Calais, France). They were transported to the Biology of Marine Organisms and Biomimetics Unit of the University of Mons, kept in a marine aquarium with closed circulation (13°C, 33‰ salinity) and fed with mussels (*Mytilus edulis* L. 1758).

### 5.2. Transmission electron microscopy

Whole tube feet were fixed by immersion in 3% glutaraldehyde in cacodylate buffer (0.1 M, pH7.8, with 1.55% NaCl) for 3 h at 4°C. The tube feet were rinsed in cacodylate buffer (0.2 M, pH7.8, with 1.84% NaCl) and then post-fixed in 1% osmium tetroxide in cacodylate buffer (0.1 M, pH7.8, with 2.3% NaCl). After rinsing in cacodylate buffer, the tube feet were dehydrated in a graded ethanol series and embedded in Spurr resin. Longitudinal semi-thin sections (1 µm) were obtained with a Reichert Om U2 ultramicrotome equipped with a glass knife. They were stained with a 1 : 1 mixture of 1% aqueous solution of methylene blue in 1% sodium tetraborate and 1% aqueous solution of azur II. Ultrathin sections (80 nm) were cut with a Leica Ultracut UCT ultramicrotome equipped with a diamond knife. They were contrasted with uranyl acetate and lead citrate and observed with a Zeiss LEO 906E transmission electron microscope.

### 5.3. Double fluorescent *in situ* hybridization

RNA probe synthesis was performed as previously described [11]. Probe sequences and primers are listed in electronic supplementary material, table S2. Briefly, transcript-specific primers were designed and a T7 promoter region was added at the 5′ end of the reverse primer. The purified PCR product was then used to produce single-stranded digoxigenin-labelled and fluorescein-labelled RNA probes (digoxigenin or fluorescein RNA labelling mix, Roche). RNA probes were used at a final concentration of 0.1–0.2 ng µl^−1^. The double ISH was performed with some alterations to the previously published protocol for paraffin section ISH [11]. The ISH signals were detected using the tyramide signal amplification (TSA) system (PerkinElmer). After blocking, sections were incubated with an Anti-digoxigenin-HRP conjugate (NEF832001EA, PerkinElmer) diluted 1 : 500 in blocking reagent (Roche) overnight at 4°C. The following day, the sections were washed 3 × 5 min in Maleic acid buffer (MAB; 100 mM Maleic acid, 150 mM NaCl, pH7.5, treated with diethyl pyrocarbonate (DEPC) and autoclaved) and 3 × 5 min in TNT buffer (0.1 M Tris HCl pH7.5, 0.15 M NaCl, 0.05% Tween-20, DEPC treated and autoclaved). Sections were incubated with Cyanine 3 plus amplification reagent (NEL753001KT, PerkinElmer) in the dark for 10 min at room temperature. After washes with TNT buffer (3 × 5 min), slides were incubated in a 1.5% solution of H_2_O_2_ in TNT buffer (3 × 20 min) and then in blocking reagent for 1 h at 4°C after washes in MAB (5 × 5 min). Slides were incubated with Anti-fluorescein-HRP conjugate (NEF710001EA, PerkinElmer) diluted 1 : 500 in blocking reagent overnight at 4°C. The following day, the sections were washed in MAB (3 × 5 min) and TNT buffer (3 × 5 min). Then, sections were incubated with fluorescein plus the amplification reagent (NEL753001KT, PerkinElmer) in the dark for 10 min at room temperature. Following several washes in TNT buffer (6 × 10 min), slides were mounted in Vectashield mounting medium with DAPI (Vector Laboratories) and analysed with an Olympus FV1000 confocal microscope.

### 5.4. Sfps *in silico* analyses

Candidate adhesive protein sequences from *A. rubens* were used as starting query sequences for tBLASTn searches in publicly available sea star transcriptomes (http://echinodb.uncc.edu) [61] and the genome of *A. rubens* (https://www.ncbi.nlm.nih.gov/genome/annotation_euk/Asterias_rubens/100/). Matching sequences were used for a reciprocal BLAST search in the *A. rubens* tube foot-specific transcriptome [6] and all sequences were used for multiple sequence alignments using the software CLC 8.0 (Qiagen). This approach allowed for the elongation of *A. rubens* sequences and elongated sequences were confirmed with PCR. The PCR products were sequenced with Sanger sequencing (Eurofins genomics) (electronic supplementary material, table S1). All sequences were translated into proteins and reanalysed with the mass spectrometry data from sea star adhesive footprints [7,8] using the software ProteinPilot 5.0 (Sciex, Singapore) (electronic supplementary material, figure S4 and electronic supplementary material, S2). Carbamidomethyl cysteine was set as fixed modification, Iodoacetamide for amino acid substitution and trypsin as digestive enzyme. Homology against known proteins was assessed using NCBI BLAST (BLASTn and BLASTp) (https://blast.ncbi.nlm.nih.gov/Blast.cgi) set to the default parameters [62]. The presence of conserved protein domains was assessed with InterPro 5.42 78.0 [63]. The presence of a signal peptide was predicted using SignalP 5.0 [64]. The amino acid composition, molecular weight and isoelectric point of each protein were determined using ProtParam from Expasy [65]. Amino acid composition biases were detected using SAPS (electronic supplementary material, table S3) [66]. NetNGlyc 1.0, NetOGlyc 4.0 and NetPhos 3.1 were used to predict N- and O-glycosylation and phosphorylation, respectively (electronic supplementary material, table S3) [67–69]. Schematic protein drawings were made with IBS software v. 1.0.3 based on InterPro results (figure 4) [70].

### 5.5. Antibody production

Three peptides were selected on the basis on their potential for successful synthesis and immunogenicity for polyclonal antibodies production in rabbits: GDSSQEIKRTLDTK (Genscript, Piscataway, NJ, USA), SLSRMDESQSTDSKL (Eurogentec, Belgium) and KMNMMTGDLVTEEY (Genscript, Piscataway, NJ, USA) from Sfp7/8, Sfp10 and Astacin-like Sfp, respectively. The Sfp7/8 peptide was present (with one aa difference: GDSSQEIKRTLDKK) in both AC-specific proteins Sfp7 and Sfp8 and therefore detects both proteins. The antibodies were isolated from the crude serum by affinity purification using the synthetic peptides (Eurogentec and Genscript).

### 5.6. Immunohistochemistry of tube foot sections

The immunohistochemistry on tube foot sections was performed with some alterations to a previously published protocol [11]. Tube feet were fixed in 4% (w/v) paraformaldehyde (PAF) in phosphate-buffered saline (PBS; pH7.4), rinsed in PBS, and subsequently dehydrated in a graded ethanol series. Tube feet were then embedded in paraffin wax and cut longitudinally into 5 µm-thick sections with a Microm HM 340 E microtome. After dewaxing and rehydration, antigen retrieval was achieved by incubation in a solution containing 0.05% (w/v) trypsin (Sigma) and 0.1% (w/v) CaCl_2_ for 15 min at 37°C. Samples were blocked in PBS containing 3% (w/v) bovine serum albumin (BSA) and 0.3% (v/v) Triton X-100 for 30 min at room temperature. The polyclonal anti-*Asterias rubens* Sfp7/8, Sfp10 and Astacin-like Sfp antibodies were diluted 1 : 100 in blocking solution and added to samples for 1 h at room temperature (see antibody production and electronic supplementary material, S2). Alexa Fluor 488-conjugated goat-anti-rabbit immunoglobulins (Invitrogen) were diluted 1 : 200 in blocking solution and applied for 1 h at room temperature. Samples were mounted in Vectashield mounting medium (Vector Laboratories) and analysed with an Olympus FV1000 confocal microscope.

### 5.7. Footprint immunolabelling

Footprints were either obtained by turning individual sea stars on their backs and letting them attach strongly to a glass slide placed on their tube feet, or by allowing sea stars to walk over a glass slide. In both cases, the sea stars and glass slides were submerged in seawater. Footprints were fixed in 4% (w/v) PAF in PBS solution, rinsed in PBS solution, dehydrated in ethanol and stored in 70% ethanol. Upon use, they were rehydrated in distilled water followed by Tris-buffered saline (25 mmol l^−1^ Tris, 125 mmol l^−1^ NaCl, pH8.0) containing 0.05% (v/v) Tween-20 (TBS-T) and submitted to the immunolabelling method detailed earlier, without the antigen retrieval step. Double immune-labelling was performed with the polyclonal anti-*Asterias rubens* Sfp1β [6] and the lectin WGA. This antibody was diluted 1 : 100 and the lectin WGA was diluted at a concentration of 25 µg ml^−1^ in TBS-T containing 3% (w/v) bovine serum albumin (TBS-T-BSA) [6,13]. Alexa Fluor 488-conjugated goat-anti-rabbit immunoglobulins (Invitrogen) were diluted 1 : 200 and Texas-Red-conjugated streptavidin (Vector Laboratories) was diluted 1 : 100 in TBS-T-BSA. Footprints were observed by using an Olympus FV1000 or Nikon TI2-E-A1RHD25 a confocal microscope.

## Data Availability

CDS sequences: Genbank accessions OP067637–OP067654. The data are provided in the electronic supplementary material [71].
